# Individualized Homeopathic Treatment for Persistent Insomnia and Generalized Anxiety Disorder: A Case Report

**DOI:** 10.7759/cureus.67203

**Published:** 2024-08-19

**Authors:** Jacqueline Soto-Sánchez, Gilberto Garza-Treviño

**Affiliations:** 1 Sección de Estudios de Posgrado e Investigación, National Polytechnic Institute, Mexico City, MEX

**Keywords:** arsenicum album, hyosciamus niger, insomnia, generalized anxiety sisorder, individualised homeopathy

## Abstract

Insomnia is a widespread disease that tends to be associated with other problems, like anxiety. The most frequent anxiety disorder is generalized anxiety disorder (GAD), which is characterized by excessive worrying about everyday situations. Medications for insomnia and anxiety can have adverse reactions and the latter may be ineffective in up to 30% of patients. Here we present a case report of a 27-year-old male patient who suffered from persistent insomnia with comorbid GAD and schizophreniform disorder. Initially, he was taking alprazolam, paroxetine, and risperidone, which had a less-than-satisfactory effect. He was treated with individualized homeopathy, which produced a remarkable improvement within four months. This was evidenced by a decrease in difficulty falling asleep and daytime sleepiness; in addition, anxiety and its accompanying symptoms, such as irritability and diaphoresis, were reduced. This improvement persists for up to one year after the commencement of treatment and despite discontinuation of all medications. This clinical report provides preliminary evidence that persistent insomnia and comorbid GAD can be treated successfully with individualized homeopathy. However, further randomized controlled studies are needed to evaluate its efficacy, effectiveness, and safety more conclusively.

## Introduction

Insomnia is a sleep disorder characterized by difficulty initiating or maintaining sleep, waking up before the desired time, and unrefreshing sleep; it can be accompanied by attention deficit and mood instability and has a minimal duration of three months [1]. Chronic insomnia has a worldwide prevalence of about 10% in the general population and 50% in patients seen in primary care [2]. Insomnia is also a frequent comorbidity of many medical, neurologic, and mental disorders, such as anxiety, depression, and schizophrenia; in fact, epidemiological studies report that insomnia affects nearly 50% of people with anxiety and schizophrenia. There is a bidirectional relationship between anxiety and insomnia: lack of sleep can cause or worsen anxiety and vice versa. Generalized anxiety disorder (GAD) is defined as excessive and uncontrollable worrying about everyday affairs, it is associated with substantial somatization and has a minimal duration of six months [3,4]. Schizophreniform disorder is an illness characterized by symptoms identical to those of schizophrenia, distinguished from it by a duration greater than one month and lesser than six months, without impairment in daily functioning [5].

The first-line treatment for insomnia is cognitive-behavioral therapy for insomnia (CBT-I) [6] and, in the case of most anxiety disorders, selective serotonin reuptake inhibitors (SSRI) and serotonin and norepinephrine reuptake inhibitors (SNRI) are employed; benzodiazepines and non-benzodiazepine receptor agonists (non-BzRA) are also used in both diseases [7]. Regrettably, these drugs are associated with several adverse reactions and 30% of patients with anxiety don’t respond to first-line medications. Treatment for schizophreniform disorder includes antidepressants and antipsychotic medications, nonetheless, many patients don’t respond to typical antipsychotics.

The essence of homeopathy, what separates it from other therapies, is the central element of its logic: the principle of similarity or the “*Simile*”. First outlined by Hippocrates and further developed by Hahnemann, it dictates that a substance can cure a sick person if the manifestations it elicits in the healthy person are similar to those of the sick one; in other words, when a substance can induce the clinical image of a pathological condition in a healthy subject, it can also, under certain circumstances, cure a similar condition in the sick person.

Studies demonstrate that, in patients with insomnia, homeopathy increases total sleep time [8], improves sleep latency [9] (the time from turning the light off to falling asleep), and sleep efficiency [10] (the ratio of total sleep time to time in bed); furthermore, the potential of homeopathy as a therapeutic alternative for the reduction of symptoms of anxiety and depression has also been demonstrated [11]; however, to date, there is a lack of research about the effect of individualized homeopathy on insomnia and other associated medical illnesses such as anxiety. Consequently, the present case study was carried out to explore the effect of individualized homeopathy on a patient suffering from persistent insomnia and GAD.

## Case presentation

Patient information

This is a 27-year-old male who came to the school clinic because of long-standing insomnia and anxiety: Insomnia, which began at age six, was characterized by difficulty initiating sleep despite having a good environment for sleeping; he took up to three hours, during which he thought and worried about academic matters and saw “abstract landscapes” (sic.); such difficulty came about in a daily fashion and it was aggravated during hot weather, by anticipation (before an exam) and anxiety. He reported nocturia (urinary frequency), which occurred two times per night, falling asleep immediately after. He also complained about non-refreshing sleep and daytime sleepiness, especially after lunch, in the late afternoon and early evening (16:00 to 18:00 hours) but didn't take naps. Regarding anxiety, it began two years earlier, after breaking up with his girlfriend, and was characterized primarily by excessive worrying about his health and secondarily about obtaining his degree; palmar diaphoresis and a sensation of heat were the main concomitants, while fatigue, irritability, increased appetite, and a craving for sweets were also associated. He also reported psychotic symptoms starting two years earlier, after the cited breakup, with auditory hallucinations (hearing multiple voices that criticized him), and visual hallucinations (shadows); moreover, he presented various events of disorganized speech followed by episodic amnesia; these symptoms ceased after five months of onset, following the prescription of an antipsychotic.

About his family history, his mother suffers from an anxiety disorder. Regarding his drug history, he was already receiving risperidone, 1 mg every 12 hours; alprazolam, 0.25 mg every eight hours; and paroxetine, 10 mg daily, prescribed by his psychiatrist; he had taken quetiapine, but it was withdrawn due to hypertriglyceridemia; he denied substance abuse or dependence. Concerning his past medical history, he accepted dyspnea and having used inhaled budesonide, and his last acute infection was seven years ago.

Clinical findings and diagnostic assessment

Physical examination revealed a hyper-resonant thorax with reduced diaphragmatic excursion to percussion, and diminished breath sounds with prolonged exhalation throughout the thorax to auscultation, findings suggestive of asthma. Still, we couldn't confirm it through spirometry. No other relevant abnormalities were found. At the first consultation and three different appointments, the patient completed three self-report scales: the Insomnia Severity Index (ISI), the Beck Anxiety Inventory (BAI), and the Hospital Anxiety and Depression Scale (HADS). The ISI is a seven-item instrument that evaluates insomnia according to widely accepted criteria, the BAI is a 21-item inventory that measures the intensity of anxiety in psychiatric populations, and the HADS is a 14-item questionnaire composed of two subscales (one for anxiety and one for depression) which exclude somatic symptoms. The three of them are used to assess treatment response in clinical practice and research.

Based on the results of the ISI, the patient was diagnosed with clinical or moderate insomnia. According to the Diagnostic and Statistical Manual of Mental Disorders, Fifth Edition (DSM-5) criteria [10], the patient had persistent insomnia: He had (A1) significant difficulty initiating sleep, which caused (B) fatigue and daytime sleepiness, this occurred in a (C) daily fashion, was (D) long-standing and (E) despite favorable circumstances; (F) he had no other sleep disorders, (G) wasn’t taking any stimulants and (H) his insomnia couldn’t be explained by anxiety, since the latter was diagnosed more recently.

The patient had a low level of anxiety according to the BAI (under allopathic treatment) and fulfilled DSM-5 criteria for a comorbid generalized anxiety disorder: (A) Excessive anxiety and worry most of the time for more than six months, which was (B) difficult to control, (C) was associated with (C2) fatigue, (C4) irritability, and (C6) insomnia, (D) caused clinically significant discomfort and (E and F) couldn’t be attributed to a substance or medical condition.

Furthermore, he met DSM-5 criteria for schizophreniform disorder: He had (A2) hallucinations and (A3) disorganized speech (B) during five months, which (C) cannot be attributed to schizoaffective disorder, major depressive disorder, bipolar disorder, (D) substance abuse, or other medical illness.

Therapeutic intervention

**Figure 1:**
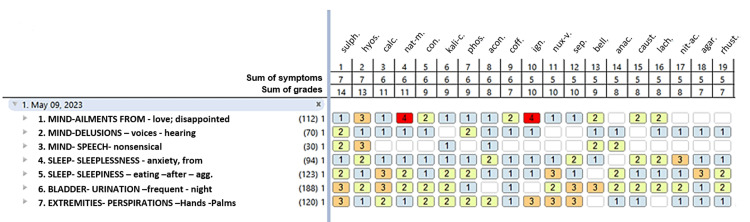
First repertorisation. Remedies are ranked by sum of symptoms. sulph: sulphur, hyos: Hyosciamus niger, calc: Calcarea carbonica, nat-m: Natrum muriaticum, con: Conium maculatum, kali-c: Kalium carbonicum, phos: phosphorus, acon: Aconitum napellus, coff: Coffea cruda, ign: Ignatia amara, nux-v: Nux vomica, sep: Sepia officinalis, bell: Belladonna atropa, anac: Anacardium orientale, caust: Causticum hahnemannii, lach: Lachesis trigonocephaluls, nit-ac: Nitricum acidum, agar: Agaricus muscarius, rhust: Rhus toxicodendron.

**Figure 2:**
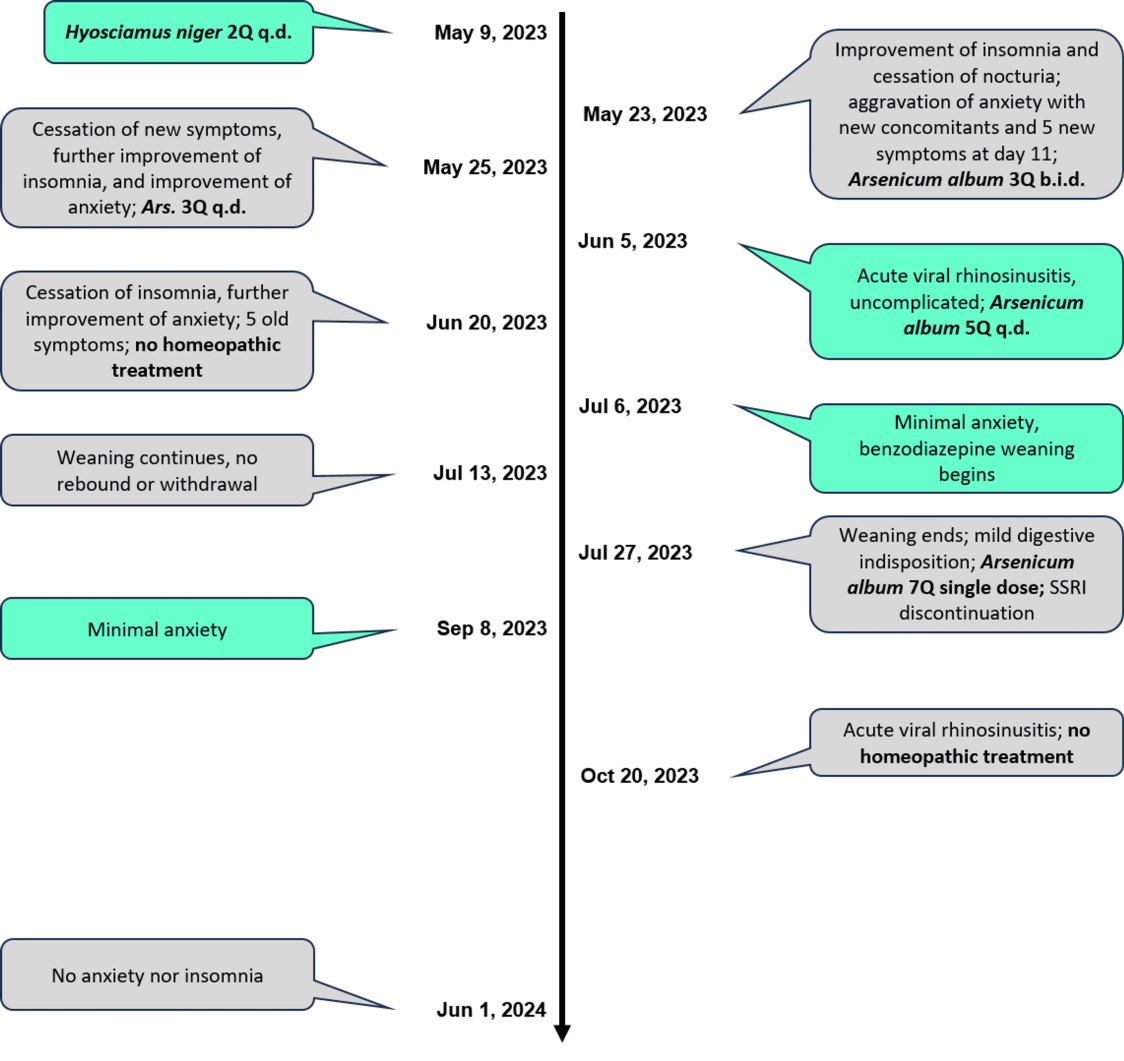
Prescriptions, key changes, and dates of all clinical encounters are depicted; clinical scales were applied at green encounters. Q: fifty-millesimal scale, q.d.: every 24 hours, b.i.d.: every 12 hours, SSRI: selective serotonin reuptake inhibitor.

According to classical homeopathy, all homeopathic prescriptions were individualized and for a single medication. The following symptoms were considered in the first meeting: the perceived cause: a disappointed love; the psychotic symptoms: auditory hallucinations (which were more frequent than the visual ones), and disorganized speech; insomnia; nocturia; anxiety and its main concomitant: palmar diaphoresis. The first repertorisation is illustrated in Figure 1. Initially (05/09/2023), the patient received *Hyosciamus niger* 2Q (fifty-millesimal scale) every 24 hours (two globules diluted in 300 ml of water, to take 10 ml each time; the bottle was succussed 10 times for every dose). All medicines were obtained from Similia® (Arrah, India). For treatment duration, see Figure 2.

Follow-up and outcomes

The main symptoms, their course, and all homeopathic prescriptions are presented in Figure 2.

**Figure 3:**
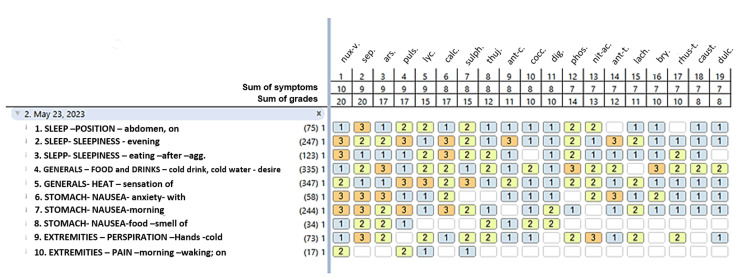
Second repertorisation. Remedies are ranked by sum of symptoms. nux-v: Nux vomica, sep: Sepia officinalis, ars: Arsenicum album, puls: Pulsatilla nigricans, lyc: Lycopodium clavatum, calc: Calcarea carbonica, sulph: sulphur, thuj: Thuja occidentalis, ant-c: Antimonium crudum, cocc: Cocculus indicus, dig: Digitalis purpurea, phos: phosphorus, nit-ac: Nitricum acidum, ant-t: Antimonium tartaricum, lach: Lachesis trigonocephalus, bry: Bryonnia alba, rhus-t: Rhus toxicodendron, caust: Causticum hahnemanni, dulc: Dulcamara solanum.

On 05/23/2023, after the first prescription, insomnia improved, falling asleep two hours earlier, and he stopped urinating at night. One week later, his psychiatrist withdrew the risperidone and decreased the dose of alprazolam to 0.25 mg every 12 hours; however, three days later, he had a panic attack which wasn't provoked by any circumstance; it was accompanied by nausea, vomiting, and dyspnea; he also reported new symptoms: epigastric pain, nausea caused by the smell of food, vesical tenesmus, decreased force of urinary stream, and myalgias and arthralgias upon awakening. *Hyosciamus niger *was withdrawn and he was started on *Arsenicum album *3Q every 12 hours; repertorisation can be consulted in Figure 3.

On 05/25/2023, two days later, insomnia and daytime sleepiness improved, as did anxiety and its concomitants, the latter having ceased; irritability and the sensation of heat also improved; and the five new symptoms also ceased. The same dynamization (3Q) was continued, decreasing the frequency to one dose every 24 hours (see Figure 2).

On 06/05/2023, insomnia improved further and fatigue ceased; he developed an acute, uncomplicated, viral rhinosinusitis. *Arsenicum album* 5Q every 24 hours was prescribed.

On 06/20/2023, the common cold subsided sooner than ordinary, it had no complications, and allopathic treatment wasn’t required. He reported minimal anxiety, and the rest of the symptoms ceased: insomnia, daytime sleepiness, the craving for sweets, and the sensation of heat. Palmar diaphoresis, previously related to anxiety, occurred separately and was brought by emotions or by seeing violence (an old symptom); additionally, four other old symptoms appeared: fear as if something bad was going to happen, flirtatiousness, a desire for cold drinks, and pain in both legs when climbing stairs. Homeopathic treatment was discontinued.

**Figure 4:**
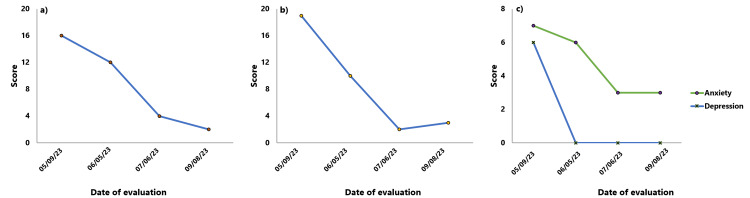
Changes in clinical scale scores over four clinical encounters: a) Variation in the Insomnia Severity Index (ISI) score, b) Variation in the Beck Anxiety Inventory (BAI) score, and c) Variation in the Hospital Anxiety and Depression Scale (HADS) score.

On 07/06/2023, significant improvement in the clinical scales was evident, as can be seen in Figure 4, and weaning of alprazolam at 0.125 mg every week was started.

On 07/27/2023, ee reported symptoms of gastroesophageal reflux disease, which subsided after a single dose of *Arsenicum album* 7Q.

On 09/08/202, alprazolam weaning lasted one month and paroxetine was withdrawn at the end; both processes were well tolerated, without rebound or withdrawal syndrome. Clinical scales were applied for the last time, showing significant improvement, as detailed in Figure 4.

On 10/20/2023, three months after stopping alprazolam and paroxetine, he remained free of insomnia and anxiety. He developed another uncomplicated acute respiratory infection, with a high fever; we prescribed chlorphenamine and acetylcysteine.

On 06/01/2024, 10 months after stopping alprazolam and paroxetine and a year after the start of the intervention, he remains free of anxiety and insomnia, and he reports no other health problems.

Adherence and adverse effects

Therapeutic adherence was optimal. We recorded one adverse event: the appearance of five new symptoms two weeks after initiation of *Hyosciamus niger*, which was inconsequential and subsided two days after changing the medicine.

Causal attribution

To assess causal attribution, we applied the Modified Naranjo Criteria for Homeopathy (MONARCH) to this case, obtaining a score of 9 points, as is shown in Table 1 [12].

**Table 1:** Modified Naranjo Criteria for Homeopathy applied to this case Criteria and their fulfillment are shown. Individual items are considered in the Discussion.

Domain		Answer	Score
1	Was there an improvement in the main symptom or condition for which the homeopathic medicine was prescribed?	Yes	2
2	Did the clinical improvement occur within a plausible timeframe relative to the medicine intake?	Yes	1
3	Was there a homeopathic aggravation of symptoms?	N/A	0
4	Did the effect encompass more than the main symptom or condition (i.e., were other symptoms, not related to the main presenting complaint, improved or changed)?	Yes	1
5	Did overall well-being improve? (Suggest using a validated scale or mention about changes in physical, emotional, and behavioral elements)	Not sure	0
6A	Direction of cure: did some symptoms improve in the opposite order of the development of symptoms of the disease?	No	0
6B	Direction of cure: did at least one of the following aspects apply to the order of improvement in symptoms:	Yes	1
	–from organs of more importance to those of less importance?		
	–from deeper to more superficial aspects of the individual?		
	–from the top downwards?		
7	Did “old symptoms” (defined as non-seasonal and non-cyclical symptoms that were previously thought to have resolved) reappear temporarily during the course of improvement?	Yes	1
8	Are there alternative causes (i.e., other than the medicine) that—with a high probability—could have produced the improvement? (Consider known course of disease, other forms of treatment, and other clinically relevant interventions)	No	1
9	Was the health improvement confirmed by any objective evidence? (e.g., investigations, clinical examination, etc.)	Yes	2
10	Did repeat dosing, if conducted, create similar clinical improvement?	N/A	0

## Discussion

According to the principles of classical homeopathy, the preferred drug should be similar to the patient’s most characteristic symptoms; by determining what is unique in a patient compared to others with the same disease, and by considering the patient as a whole, homeopaths direct the treatment not only to the main complaints but to the individual combination of pathological processes and their expression, neutralizing their root cause.

In the present paper, we present the case of a young man with a diagnosis of persistent insomnia with comorbid GAD and schizophreniform disorder. He was prescribed *Hyosciamus niger* due to its similarity with the outermost symptom layer: anxiety and psychosis (his most recent diagnoses), their perceived cause, and insomnia, (a chronic condition, but modified by the former). Psychotic symptoms were taken into account due to their temporal association with anxiety and their triggering by the same event; this is in accordance with a homeopathic precept which dictates that symptoms should be taken as they were before allopathic treatment.

After the administration of *Hyosciamus niger*, there was a notable improvement in symptoms such as difficulty initiating sleep and nocturia. In this regard, *Hyosciamus niger *is known to contain hyoscyamine, scopolamine, and anisodamine [13]; these substances act as competitive antagonists of muscarinic acetylcholine receptors, which potently modulate the central nervous system. Hyoscyamine has been used, in conventional medicine, to treat symptoms related to overactive bladder [14]; this may explain the observed reduction of nocturia in our patient. On the other hand, homeopathy is based on the “law of similars” [15], which means that a substance that causes certain symptoms in a healthy individual can be employed to treat analogous symptoms in a sick person. In this context, it's noteworthy that hyoscyamine and scopolamine can cause potent psychotropic effects, including characteristic delirium-like states with hallucinations, mood disturbances, and cognitive deficits [16]. Additionally, hyoscyamine is the levorotatory isomer of atropine; atropine has been found to increase rapid eye movement (REM) sleep in cats with serotonin synthesis inhibitor-induced insomnia, but reduce REM sleep in normal cats, suggesting an interaction between serotonin and acetylcholine [17].

Continuing with the patient’s evolution, after two weeks of continued improvement, an aggravation with new symptoms occurred: these symptoms have been reported for *Hyosciamus niger *in several *materia medica*. This does not correspond with any of Kent’s [15] observations (his fifth observation does not include new symptoms, the eighth observation does not include improvement, and the 10th observation does not include improvement nor aggravation); but it would be explained by Vithoulkas’ [18] fourth observation, considering that a premature alprazolam dose reduction caused the worsening of anxiety. Vithoulkas’ fourth observation states that a correct prescription can produce new symptoms in a susceptible or hypersensitive patient. In the same vein, the advent of new symptoms could have indicated the dissimilarity of *Hyosciamus niger* with the clinical picture of the patient at that moment. Finally, it was concluded that the clinical picture had changed and gave him a different remedy.

Two weeks after starting the *Arsenicum album*, the appearance of an acute respiratory infection, of short duration, mild, and uncomplicated (contrary to what would be expected in a patient with a history of bronchial hyperresponsiveness), confirmed that the prescription was correct according to Vithoulkas’ [18] theory: His level of health increased to the seventh. The authors believe that the patient rose to level six, entering group B, four months later when he developed another acute respiratory infection, this time with fever. Similarly, five old symptoms emerged one month after starting the *Arsenicum album*, corresponding with Kent’s [15] 11th observation. In conjunction with the patient's multidimensional improvement, these observations lead us to conclude that the prescription was correct. *Arsenicum album* treatment was highly efficacious in alleviating the remaining symptoms.

*Arsenicum album* is prepared from arsenic trioxide (As2O3); at doses of 15 mg, this compound induces anxiety-like behaviors in mice, which are mediated by altered GABAergic signaling in the prefrontal cortex [19]; however, at the homeopathic level (ultra-diluted doses), it has been reported to alleviate anxiety [20]. This study provides reasonable clinical evidence for the design of randomized controlled studies to evaluate the efficacy, effectiveness, and safety of individualized homeopathy for the treatment of persistent insomnia and comorbid anxiety.

Respecting the MONARCH, item 1 was answered "Yes" due to evident improvement of the main symptoms, such as difficulty falling asleep, anxiety about his health, palmar diaphoresis, and the sensation of heat. Item 2 was responded to with "Yes", as clinical improvement occurred within days following all prescriptions. Item 3 addresses the homeopathic aggravation, which didn't occur: it may seem that the panic attack experienced by the patient after the first prescription is an aggravation, but it happened 11 days after the first dose of *Hyosciamus niger*, which is belated according to Kent [15]; similarly, the said attack can be attributed to an untimely decrease in the dose of alprazolam; finally, a homeopathic aggravation is not expected with the posology applied to this case: thus, item 3 was answered "N/A". Item 4 concerns the multidimensionality of improvement, characteristic of a successful homeopathic prescription; it was responded to as "Yes" because the symptoms not related to his diseases, such as nocturia, nightmares, and a craving for sweets subsided; similarly, his oxygen saturation, as measured by pulse oximetry, went from 89 to 95%. Item 5, which refers to improvement in quality of life, was answered "Not sure"; this is because the quality of life wasn't measured objectively, but the authors think it did improve since his physical, emotional, and behavioral spheres showed favorable changes. Item 6A relates to the direction of cure: specifically, if symptoms improved in the opposite order of their development; it was responded to with "no", since the main complaints: anxiety and insomnia, subsided simultaneously. Closely related, item 6B also pertains to the direction of cure, which went from organs of more importance to those of less importance: from the central nervous system to the musculoskeletal and digestive systems; thus, it was answered "Yes". Item 7 was responded to with "Yes" due to the advent of five old symptoms after *Arsenicum album *5Q. Item 8 was answered "No" since no additional interventions were prescribed by his psychiatrist or us, including lifestyle changes. Item 9 was responded to with "Yes", as three validated scales were used to measure improvement. Finally, item 10 is not applicable because his symptoms haven't recurred.

## Conclusions

This case of persistent insomnia with comorbid GAD was successfully treated with individualized homeopathy, as evidenced by our clinical assessment and the clinical scales applied: the patient started with mild anxiety, according to the BAI, under allopathic treatment and ended with an even lower level and without psychotropic drugs; moreover, the patient's insomnia level, which was initially moderate despite taking two somnifacients, decreased to a non-significant level, even after he quitted the aforementioned drugs. These therapeutic effects of homeopathic treatment persist to the day the authors are writing this conclusion. Further clinical research with an appropriate sample size, evaluating the individualized approach in cases of persistent insomnia with comorbid anxiety is needed to confirm our results.
